# Effect of neuromuscular blockade reversal strategy on gastrointestinal recovery following abdominal wall reconstruction: a randomized controlled trial

**DOI:** 10.1007/s10029-026-03814-4

**Published:** 2026-08-07

**Authors:** Daphne Remulla, Noah X. Tocci, Ryan C. Ellis, William C. Bennett, Alvaro Carvhalo, Chao Tu, Alparslan Turan, Luciano Tastaldi, Ajita S. Prabhu, David M. Krpata, Lucas R. Beffa, Benjamin T. Miller, Michael J. Rosen, Clayton C. Petro

**Affiliations:** 1 Department of Surgery, Cleveland Clinic Foundation, 9500 Euclid Ave., Cleveland, OH 44195 USA; 2 Cleveland Clinic Center for Abdominal Core Health, Cleveland Clinic Foundation, Cleveland, OH USA; 3 Department of Biostatistics, Cleveland Clinic Foundation, Cleveland, OH USA; 4 Center for Outcomes Research, Department of Anesthesiology and Pain Medicine, University of Texas, Houston, USA; 5 Cleveland Clinic Lerner College of Medicine of Case Western Reserve University School of Medicine, Cleveland, OH USA; 6 Department of Surgery, Northwestern Feinberg School of Medicine, Chicago, IL USA

**Keywords:** Abdominal wall reconstruction, Ileus, Neuromuscular blockade reversal, Sugammadex

## Abstract

**Purpose:**

Evaluate the impact of sugammadex on gastrointestinal recovery following complex AWR.

**Methods:**

This single-center, double-blinded, randomized controlled trial randomized 184 patients undergoing open retromuscular AWR to receive either sugammadex or neostigmine/glycopyrrolate for neuromuscular blockade reversal per drug packaging protocol. The primary outcome was time to gastrointestinal recovery (GI-2), defined as tolerance of solid food and first bowel movement and univariate and multivariate analysis was used to compare sugammadex and neostigmine/glycopyrrolate cohorts. Secondary outcomes included incidence of POI, hospital length of stay, and opioid consumption among our two cohorts.

**Results:**

The final analysis included 177 patients (88 sugammadex, 89 neostigmine/glycopyrrolate). Median time to GI-2 recovery was similar between groups (89 vs. 87 h, Wilcoxon rank-sum *p* = 0.637). No significant differences were observed in POI incidence (21% vs. 25%, *p* = 0.52) or length of stay (5 vs. 4 days, *p* = 0.90). Multivariable analysis confirmed sugammadex did not significantly affect time to GI-2 recovery (HR 0.96, 95% CI 0.69–1.32, *p* = 0.79). Independent predictors of delayed recovery included POI development and greater opioid consumption.

**Conclusions:**

Sugammadex did not result in faster return to gastrointestinal function when compared with neostigmine/glycopyrrolate when used for neuromuscular blockade reversal. Thus, avoidance of neostigmine/glycopyrrolate did not reduce POI incidence or hospital length of stay in patients undergoing complex AWR.

## Introduction

Postoperative ileus (POI) is an iatrogenic condition characterized by slowed or arrested intestinal motility due to nonmechanical factors after surgery [1]. Postoperative ileus (POI) is one of the most common complications following major abdominal surgery and is consistently associated with substantial adverse effects, including prolonged hospitalization, increased healthcare costs, and substantial patient discomfort from abdominal distension, nausea, and intolerance of oral intake [2, 3]. While rates of postoperative ileus range from 5% to 25% following abdominal surgery, more specifically, in the context of complex open abdominal wall reconstruction (AWR), POI is noted to range from 13.2% to 20%, with affected patients experiencing substantially longer hospital stays (8.4 ± 3.5 days versus 5.7 ± 3.2 days) and greater healthcare costs [1, 2].

Multiple strategies to reduce postoperative ileus have been proposed, many of which have been incorporated into enhanced postoperative recovery protocols [4, 5]. Recent investigations have focused on neuromuscular blockade reversal agents as potential contributors to POI development [4, 6, 7]. Neostigmine/glycopyrrolate reversal may delay gastrointestinal recovery because the antimuscarinic effects of glycopyrrolate counteract neostigmine-induced cholinergic stimulation of gut M2/M3 receptors and the cholinergic anti-inflammatory pathway, mechanisms that otherwise promote resolution of postoperative ileus [7, 8]. In contrast, sugammadex offers an alternative mechanism through selective cyclodextrin encapsulation of neuromuscular blocking agents, achieving complete reversal without cholinergic interference [9]. This mechanism theoretically preserves normal gastrointestinal motility and may reduce POI incidence. However, existing literature on gastrointestinal outcomes demonstrates mixed results, particularly in complex abdominal surgery populations, and no studies have specifically evaluated sugammadex’s impact on POI following complex AWR [10, 11].

We therefore designed a randomized controlled trial with the aim of investigating whether sugammadex would reduce time to gastrointestinal recovery after open AWR for patients with ventral hernias < 20 cm in width, compared to standard neostigmine/glycopyrrolate reversal. Our secondary objectives included the incidence of postoperative ileus requiring nasogastric tube placement, hospital length of stay and opioid consumption. We hypothesized that sugammadex would result in faster return to gastrointestinal function when compared with neostigmine/glycopyrrolate neuromuscular blockade reversal.

## Methods

### Study design and oversight

This was a double-blinded, randomized controlled parallel group trial with patients randomized to sugammadex or neostigmine/glycopyrrolate for paralytic reversal. The Abdominal Core Health Quality Collaborative (ACHQC) and Research Electronic Data Capture (REDCap) database served as data collection platforms. All patients underwent open AWR with retromuscular mesh, performed by staff surgeons at a single tertiary academic center (Cleveland Clinic Foundation, Cleveland, Ohio). Institutional review board approval was granted prior to enrollment (IRB #23–700), and all study participants provided written informed consent. The study was registered on ClinicalTrials.gov (NCT05985343) prior to patient enrollment and conducted according to the Consolidated Standards of Reporting Trials (CONSORT) guidelines for randomized controlled trials.

### Patients and study setting

All patients with ventral hernias requiring at least one myofascial advancement flap presenting to our center were screened for enrollment by treating physicians or qualified research personnel. Inclusion criteria were adult patients ≥ 18 years old undergoing open retromuscular ventral hernia repair with mesh and myofascial release for hernias with fascial defects < 20 cm in width based on preoperative imaging.

Exclusion criteria were known hypersensitivity reactions to sugammadex or its components, hernias with fascial defects ≥ 20 cm in width, known or suspected small bowel obstruction at the time of repair, presence of nasogastric, orogastric, or gastric tubes at the time of surgery, malignancies with increased risk of gastroduodenal perforation, gastrointestinal disorders affecting intestinal integrity (e.g., Crohn’s disease), severe hepatic failure (Child-Pugh Class C) or clinically relevant hepatic impairment, severe renal failure (GFR < 30 mL/min, dialysis dependence, or presence of vascular access for renal replacement therapy), pregnancy or breastfeeding, other contraindications to sugammadex, inability to provide informed consent, vulnerable populations, non-English speaking patients, emergent operations, minimally invasive approaches, mesh placement in non-retromuscular positions, and chronic opioid use (defined as daily opioid use for ≥ 90 days within the past year or surgeon discretion). Patients who were enrolled but determined by the attending anesthesiologist to be clinically unsuitable for randomization at the time of surgery were excluded, and therefore not randomized to the intervention.

#### Interventions

On the day of surgery, general anesthesia was induced per the discretion of the anesthesiologist using propofol or etomidate, fentanyl, and rocuronium (0.6 mg/kg for intubation, 0.15 mg/kg for maintenance). Anesthesia was maintained with sevoflurane or isoflurane, along with opioids and rocuronium as clinically indicated. Intraoperative analgesia was limited to fentanyl or hydromorphone. Neuromuscular monitoring was performed by the anesthesia team. Postoperatively, patients were managed according to a standard institutional enhanced recovery after surgery (ERAS) which includes employing early mobilization and per os intake on postoperative day 0 following recovery from the anesthesia unit. Additionally, all patients were placed on multimodal pain regimen including a patient controlled intravenous analgesia pump, acetaminophen, topical lidocaine patches, and the addition of a muscle relaxant in select patients, as well as an antiemetic agent. Deviations from the institutional ERAS pathway were noted when occurring.

Randomization was performed by a study coordinator, who then delivered the appropriate treatment to the anesthesia team. Both the surgical team and patient were blinded to allocation. Upon randomization, patients received either intravenous sugammadex (2 mg/kg for ≥ 2 twitches or 4 mg/kg for 1–2 post-tetanic twitches according to package insert recommendations) or standard neostigmine/glycopyrrolate (0.03–0.07 mg/kg neostigmine with fixed-ratio glycopyrrolate).

### Data collection

Patients remained blinded to their randomization postoperatively. Research personnel visited hospitalized patients daily starting on postoperative day 1 and continuing until first bowel movement, tolerance of solid food, or postoperative day 4, whichever occurred last. In cases where patients were discharged prior to their first bowel movement and/or postoperative day 4, research personnel contacted patients by telephone with a maximum of 3 phone call attempts per postoperative day. Patient demographics, medical comorbidities, and operative details were recorded in the ACHQC database per standard institutional practice. Supplemental data not captured by ACHQC were collected by research staff and stored in REDCap, including detailed operative timing, extent of adhesiolysis, POI and daily opioid consumption. Notably, the extent of adhesiolysis was determined at surgeon discretion and case-specific, with binary assessment of whether a complete adhesiolysis occurred and the duration of adhesiolysis recorded for each case. All 30-day postoperative data were prospectively collected as standard of care, including length of stay, readmissions, reoperations, and other complications.

### Outcomes

The primary outcome was time to gastrointestinal recovery of function (GI-2), which was a composite variable of solid food tolerance and first bowel movement, representing global return of gastrointestinal function. This standardized composite endpoint is widely validated in postoperative ileus research and reflects clinically meaningful recovery across both upper and lower gastrointestinal tract function. Within this composite variable, diet toleration was defined as resuming a solid food diet without associated nausea or emesis requiring intervention such as nasogastric tube or a reduction in diet order (i.e. a liquid diet or nil per os). Time to diet toleration was defined by hours from surgery completion to solid food diet ordered in the electronic medical record (EMR) which was monitored through reports in the electronic medical record and confirmed by research coordinator assessments on daily rounds. At our institution, multidisciplinary rounds occur numerous times a day, with evaluation of patients’ postoperative progress during each rounds. When a diet order is changed, a patient has the ability to submit a tray request for food with nominal delay from order time to tray delivery, with additional patient snacks that are in accordance with a patient’s diet order provided by nursing staff between meal times. Return of bowel function was defined as passage of a bowel movement, which was assessed through EMR reports and confirmed by research coordinators on daily rounds. This was also measured in hours since surgery completion, as documented in the EMR and confirmed by research coordinators. If a patient did not have return of bowel function prior to discharge from the hospital, patients were called by research coordinators to assess return of bowel function.

Secondary outcomes included incidence of postoperative ileus, as defined as a clinically meaningful arrest of bowel function requiring the insertion of a nasogastric tube for decompression. This definition serves as an objective and clinically meaningful indicator of gastrointestinal dysfunction as it captures only events necessitating intervention. Additional secondary outcome measures included total hospital length of stay and cumulative opioid consumption measured by morphine milligram equivalents (MME).

### Power calculation

Our study investigated the superiority of sugammadex in reducing time to return of gastrointestinal function compared to neostigmine/glycopyrrolate. Based on preliminary institutional data showing median time to GI-2 function of 76.6 h (interquartile range 65.1–108.3), consensus opinion among investigators was that a 35% reduction (~ 1 day) would represent a clinically meaningful finding. At 80% power while holding type I error at 5%, a sample size of 184 total patients with 92 in each arm was required, corresponding to a hazard ratio of 1.54.

### Randomization and blinding

Randomization was generated by an institutional statistician, and allocation was completed by research coordinators. Eligible patients were randomized consecutively in a 1:1 fashion using a central concealed, random-block randomization scheme housed in REDCap. A study coordinator applied the allocated intervention. The surgical team and patients remained blinded to randomization throughout the trial.

In our original study design, randomization occurred at induction of anesthesia. However, several participants were identified as screen failures intraoperatively when the intended retromuscular abdominal wall reconstruction was not feasible. To prevent randomization of ineligible cases, we revised the timing of randomization early in the study to occur only after the operating surgeon confirmed that the planned retromuscular repair would proceed as intended. This procedural modification maintained allocation concealment while reducing post-randomization exclusions.

### Statistical analysis

Randomized groups were assessed for balance on baseline characteristics using standardized differences, defined as the difference in means or proportions divided by the pooled standard deviation. An absolute standardized difference > 0.10 was considered imbalanced. Analysis was performed under the principles of intention-to-treat with no interim analyses planned.

The primary outcome, time to GI-2 function (tolerance of solid food and first bowel movement), was analyzed as a time-to-event variable. Kaplan–Meier methods were used to estimate cumulative incidence curves, and groups were compared using the log-rank test. The median time to GI-2 with interquartile range (IQR) was reported for each group. Cox proportional hazards regression was used to estimate hazard ratios (HRs) with 95% confidence intervals (CIs) for sugammadex versus neostigmine/glycopyrrolate, adjusting for prespecified covariates (age, sex, duration of adhesiolysis, operative time, intraoperative enterotomy or bowel resection, postoperative ileus, and total opioid consumption within the first 48 h postoperatively).

Secondary outcomes were analyzed using appropriate statistical tests: two-sample t-tests or Wilcoxon rank-sum tests for continuous variables and chi-square or Fisher’s exact tests for categorical variables. No formal multiplicity adjustment was applied for secondary endpoint comparison. All analyses were performed using SAS software (version 9.4) or R software (version 4.0.0). *P* < 0.05 was considered significant.

## Results

### Patients

**Fig. 1:**
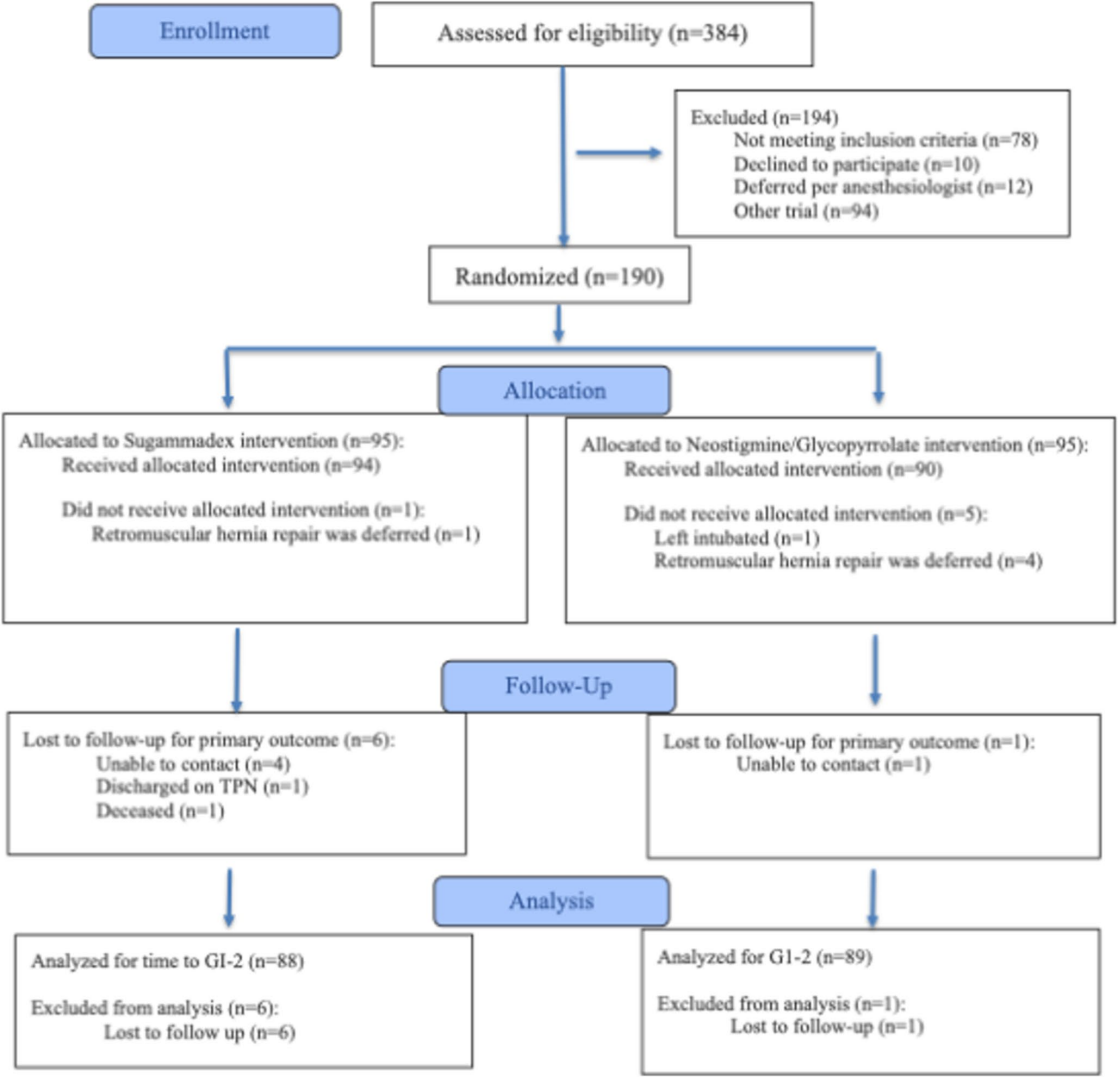
CONSORT Flow Diagram

Enrollment commenced in January 2024 and concluded in June 2025 due to trial completion. Although powered for 184 participants, enrollment was extended to 190 to account for post-randomization screen failures identified early in the study. The CONSORT analysis documenting the flow of participants from randomization through the completion of follow-up is illustrated in Fig. 1.

Of 384 patients assessed for eligibility, 194 were excluded (Fig. 1.). A total of 190 patients were randomized (95 per group). Six patients did not receive the allocated intervention as a result of the retromuscular repair being deferred intraoperatively or the patient remained intubated postoperatively due to surgeon’s discretion (1 sugammadex, 5 neostigmine/glycopyrrolate). Seven participants lacked primary outcome data with the final analysis including 177 patients: 88 in the sugammadex group and 89 in the neostigmine/glycopyrrolate group. Within this cohort, all but 15 (10 sugammadex, 5 neostigmine/glycopyrrolate) were initiated on an institutional ERAS. Non-initiation of ERAS protocol was selected at surgeon preference, which was noted to be due to extensive surgical adhesiolysis in 12 cases, post-extubation respiratory insufficiency requiring non-invasive positive pressure ventilation in 1 case, and postoperative hypotension requiring intensive care monitoring in 2 patients.

Patient demographics and operative details were well balanced between groups (Table 1). Median age was 64.0 years (IQR 54.0–70.8), and 88 participants (47.8%) were male. The most common comorbidities were hypertension (130, 70.7%) and diabetes (44, 23.9%). Median BMI was 32.0 kg/m² (IQR 27.4–35.8). Most patients were ASA Class 3 (148, 80.4%), and the majority of procedures were clean cases (153, 83.2%). Median hernia width was 15.0 cm (IQR 11.0–17.0). Operative time ranged from 120 to 179 min in most cases (81, 44.0%). Complete interloop adhesiolysis was performed in the majority of patients (131, 73.6%).

**Table 1:** Patient Demographics and Operative Characteristics

	Overall (*N* = 184)	Sugammadex (*N* = 94)	Neostigmine/Glycopyrrolate (*N* = 90)
Age (median [IQR])	64 [54, 71]	64 [54, 69]	64 [55, 71]
Male Gender, N (%)	88 (48)	40 (43)	48 (53)
Race, N (%)			
White	160 (87)	79 (84)	81 (90)
Black or African American	10 (5)	7 (7)	3 (3.3)
Other or unknown	14 (8)	8 (9)	6 (7)
Functional Status, N (%)			
Independent	171 (93)	89 (95)	82 (91)
Partially Dependent	8 (4)	4 (4)	4 (4)
Unknown	5 (2.7)	1 (1.1)	4 (4.4)
BMI, (median [IQR])	32.0 [27.4, 35.8]	31.60 [27.3, 34.8]	32.3 [27.7, 37.2]
Liver Failure, N (%)	3 (2)	2 (2)	1 (1)
Hypertension, N (%)	130 (71)	65 (69)	65 (72)
Diabetes, N (%)	44 (24)	20 (21)	24 (27)
COPD, N (%)	17 (9)	7 (7)	10 (11)
Dyspnea, N (%)	22 (12)	10 (11)	12 (13)
Antiplatelet Use, N (%)	10 (5)	6 (6)	4 (4)
Anticoagulation Use, N (%)	18 (10)	13 (14)	5 (6)
Steroid Use, N (%)	14 (8)	8 (9)	6 (7)
Nicotine Use, N (%)	17 (9)	9 (10)	8 (9)
Heart Failure, N (%)	11 (6)	7 (7)	4 (4)
ASA Class			
1	2 (1)	2 (2)	0 (0)
2	31 (17)	19 (20)	12 (13)
3	148 (80)	71 (76)	77 (86)
4	3 (2)	2 (2)	1 (1)
Wound Class, N (%)			
Clean	153 (83)	79 (84)	74 (82)
Clean-contaminated	27 (15)	13 (14)	14 (16)
Contaminated	3 (2)	1 (1)	2 (2)
Dirty/Infected	1 (1)	1 (1)	0 (0)
Operative Time, N (%)			
0–59	1 (1)	0 (0)	1 (1)
60–119	25 (14)	10 (11)	15 (17)
120–179	81 (44)	43 (46)	38 (42)
180–239	50 (27)	28 (30)	22 (24)
240+	27 (15)	13 (14)	14 (16)
Intraoperative Rocuronium (mg) use	120 [100–150]	127 [100–150]	113 [100–140]
Intraoperative Fluid (L) use	2 [1.5–2.5]	2 [1.5–2.5]	1.9 [1.4–4.0]
Hernia Length (median [IQR])	22.0 [18.0, 25.0]	23.0 [18.3, 25.0]	21.5 [18.0, 25.0]
Hernia Width (median [IQR])	15.0 [11.0, 17.0]	13.0 [11.0, 16.0]	15.0 [12.0, 17.0]

### Primary outcome

**Fig. 2:**
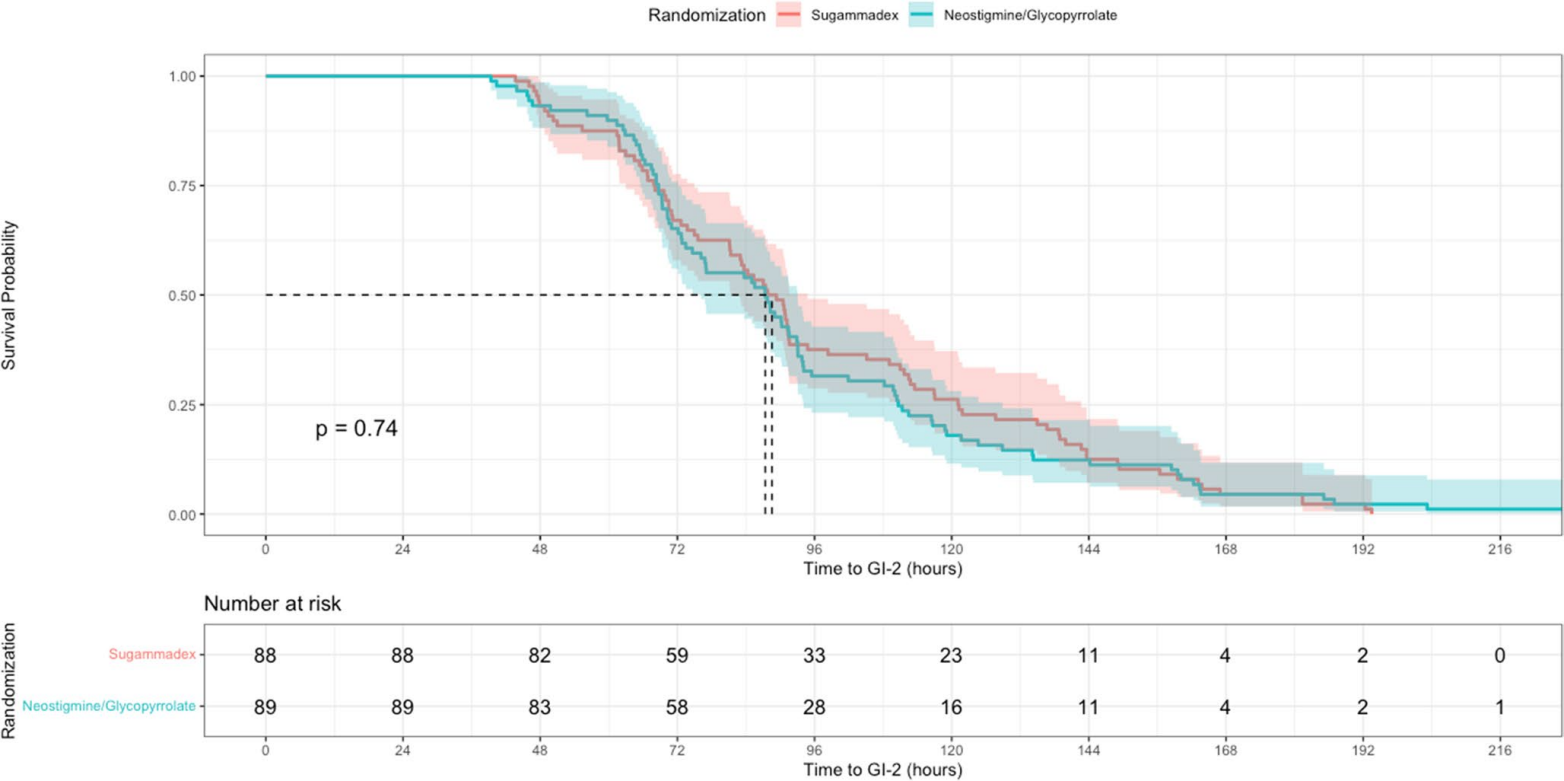
Kaplan-Meier Analysis of Time to Gastrointestinal Recovery

Median time to GI-2 recovery was similar between groups (Sugammadex: 89 h [IQR 68–121] vs. Neostigmine/Glycopyrrolate: 87 h [IQR 69–111]; Wilcoxon rank-sum *p* = 0.637, (Table 2)). Kaplan–Meier analysis demonstrated no significant difference in time to GI-2 recovery between groups (log-rank *p* = 0.74, (Fig. 2)). In multivariable Cox proportional hazards modeling adjusting for sex, operative time, duration of adhesiolysis, and intraoperative enterotomy or bowel resection, sugammadex was not associated with a significant difference in time to GI-2 recovery compared with neostigmine/glycopyrrolate (HR 0.92; 95% CI 0.67–1.26; *p* = 0.61, (Table 3)). Patient sex, operative time, duration of adhesiolysis, and intraoperative enterotomy or bowel resection, were not significantly associated with time to GI-2 recovery (Table 3). In exploratory analyses including postoperative opioid exposure, higher consumption of opioids at 48-hour postoperatively was associated with prolonged time to GI-2 recovery (HR per 100 MME: 0.90; 95% CI 0.83–0.98; *p* = 0.015). Reversal strategy remained unassociated with GI-2 recovery in this exploratory model (HR 1.04; 95% CI 0.75–1.45; *p* = 0.796). The proportional hazards assumption was met for the primary model based on scaled Schoenfeld residuals.

**Table 2:** Postoperative Outcomes

Variable	All (*n* = 177)	Sugammadex (*n* = 88)	Neostigmine/Glycopyrrolate (*n* = 89)	*p*-value
Time to GI-2 recovery (hours)	88 [68, 117]	89 [68, 121]	87 [69, 111]	0.637
Postoperative Ileus, N (%)	41 (23%)	18 (21%)	23 (25%)	0.52
Hospital length of stay (days)	5 [4, 6]	5 [4, 6]	4 [4, 6]	0.903
Duration of Adhesiolysis (hours)	0.5 [0.3, 1.1]	0.6 [0.4, 1.1]	0.5 [0.3, 0.9]	0.167
POD 1 opioid use (MME)	122.5 [52.5, 196.8]	121.2 [57.5, 200.6]	122.5 [52.5, 190.2]	0.59
POD 2 opioid use (MME)	37.5 [7.5, 92.1]	40.0 [9.6, 108.9]	35.0 [7.5, 72.5]	0.475
POD 3 opioid use (MME)	17.5 [0.0, 45.0]	15.0 [0.0, 47.4]	20.0 [0.0, 37.5]	0.923
POD 4 opioid use (MME)	9.9 [0.0, 36.2]	15.0 [0.0, 42.5]	5.0 [0.0, 22.5]	0.009

P-values are calculated using two-sample t-tests or Wilcoxon rank-sum tests for continuous variables and chi-square or Fisher’s exact tests for categorical variables

**Table 3:** Multivariate Analysis

Variable	HR	95% CI	*p*-value
Sugammadex vs. Neostigmine/Glycopyrrolate	0.92	0.67–1.26	0.61
Male sex	0.93	0.68–1.27	0.66
Operative time > 120 min	1.25	0.81–1.94	0.31
Duration of adhesiolysis	1.00	0.31–3.20	1.00
Intraoperative enterotomy or bowel resection	1.16	0.56–2.39	0.69

### Secondary outcomes

Secondary outcomes are detailed in Table 2. Median opioid consumption decreased progressively each postoperative day: 122 MME on day 1 [IQR 52.5–197], 37.5 MME on day 2 [IQR 7.5–92.1], 17.5 MME on day 3 [IQR 0.0–45.0], and 9.9 MME on day 4 [IQR 0.0–36.2]. There were no differences in opioid consumption between groups on postoperative day 1 (*p* = 0.59), day 2 (*p* = 0.48), or day 3 (*p* = 0.92). However, patients who received sugammadex reported higher opioid consumption on postoperative day 4 compared to the neostigmine/glycopyrrolate group (15.0 MME [IQR 0.0–42.5] vs. 5.0 MME [IQR 0.0–22.5]; *p* = 0.009).

The overall postoperative ileus rate was 23.2%. There was no difference between reversal agent groups (sugammadex: 20.7% vs. neostigmine/glycopyrrolate: 24.5%, *p* = 0.52). This finding was confirmed on multivariate logistic regression adjusting for relevant covariates (OR 0.71; 95% CI 0.34–1.49; *p* = 0.37).

The median length of stay for the cohort was 5 days [IQR 4–6]. There was no significant difference between groups (sugammadex: 5 days [IQR 4–6] vs. neostigmine/glycopyrrolate: 4 days [IQR 4–6]; *p* = 0.90). Multivariate analysis confirmed that reversal agent was not significantly associated with length of stay (β = 0.54, *p* = 0.34). Development of postoperative ileus was a strong predictor of prolonged length of stay, with an estimated increase of 2 days compared to patients without ileus (β = 2.18, *p* = 0.002). Greater 48-hour opioid use (β = 0.0064 per MME unit, *p* < 0.001) was also significantly associated with longer length of stay.

## Discussion

In this single-center, double-blinded, randomized controlled trial, time to return of gastrointestinal function was no different in patients undergoing open abdominal wall reconstruction who received sugammadex compared to those who received neostigmine/glycopyrrolate. Furthermore, there was no benefit in reducing incidence of postoperative ileus or hospital length of stay. These findings suggest that despite theoretical advantages and pharmacological superiority in neuromuscular reversal, the use of sugammadex does not translate to clinically meaningful improvements in gastrointestinal recovery for this specific surgical population.

The theoretical rationale for expecting superior gastrointestinal outcomes with sugammadex appears sound. Unlike neostigmine/glycopyrrolate, which increases acetylcholine at muscarinic receptors and requires concurrent anticholinergic administration, suppressing gut motility, sugammadex reverses neuromuscular blockade without affecting cholinergic pathways. This mechanism in theory preserves physiologic peristalsis and may support gastrointestinal recovery [12]. Despite this, these findings have not translated into clinically meaningful benefits [6–8, 13], and pooled analyses and randomized controlled trials consistently demonstrate no significant difference in postoperative ileus or length of stay based on anesthetic reversal strategies [9, 10, 14]. This pattern likely reflects the multifactorial pathophysiology of postoperative gastrointestinal recovery, which involves complex interactions between neurogenic reflexes, inflammatory cascades, hormonal responses, and surgical trauma [15, 16]. With the overall POI rate of 23.2% and median time to recovery of GI-2 function of 87.7 h reflect the substantial burden of delayed gastrointestinal recovery in the AWR population. Several factors unique to AWR patients may predispose this population to particularly high POI rates independent of neuromuscular blockade reversal, namely extensive bowel manipulation, inflammatory response from mesh implants, and narcotic use for postoperative analgesia. In our cohort, greater opioid consumption was significantly associated with delayed GI-2 function, consistent with the well-established mechanism of opioid-induced gut dysmotility through peripheral µ-opioid receptor activation [12]. The lack of improvement in GI recovery with sugammadex, despite adequate statistical power and rigorous methodology, provides important evidence that neuromuscular blockade reversal choice may not be a significant modifiable factor for POI in complex abdominal procedures. Given the extensive inflammatory response inherent to complex AWR, the multifactorial pathophysiology of POI likely overwhelms any marginal benefits from optimizing neuromuscular reversal strategies.

Our findings have important implications for clinical practice and healthcare economics. The average cost of sugammadex reversal is estimated to be over 5 times greater than neostigmine/glycopyrrolate, with representative pricing showing sugammadex at $148–271 per vial compared to $13.50 for neostigmine/glycopyrrolate combination [17]. However, recent evidence suggests the total cost impact is more nuanced than drug acquisition costs alone. In a large multicenter study of 79,474 surgical patients, Wachtendorf et al. found that sugammadex was associated with $232 lower total hospital costs overall, but this effect was highly dependent on patient risk profile: low-risk patients (ASA 1–2 undergoing ambulatory surgery) experienced $1,042 lower costs with sugammadex, while high-risk patients (ASA ≥ 3 with preoperative hospitalization) had $620 higher costs [18]. Without demonstrated clinical benefits in high-risk AWR populations, routine sugammadex use could substantially increase healthcare expenditures without improving outcomes, particularly in high volume and high complexity abdominal wall reconstruction centers. Our findings suggest that sugammadex benefits are likely procedure-specific and population-dependent, with advantages most apparent in ambulatory and low-risk settings. In these contexts, where residual neuromuscular blockade significantly contributes to complications and faster recovery times translate to operational efficiency, both the pharmacological and economic benefits of sugammadex may be realized [19, 20].

This study has several limitations. First, due to the initial study design, we experienced several screen failures and missing primary-outcome data. Although enrollment was extended to 190 to offset post-randomization exclusions, minor group imbalance and a 3.7% rate of missing outcomes may have reduced analytic power. Additionally, our primary outcome GI-2 of diet tolerance relies on electronic medical record order time which may not adequately capture the clinical circumstances of a patient’s postoperative course. Second, because anesthesiologist participation was required for each case, randomization occasionally depended on staff approval to not use neostigmine/glycopyrrolate, which may have introduced pragmatic variability in group allocation. Additionally, reliance on neuromuscular monitoring by the anesthesia team through qualitative measures may introduce a source of misclassification of twitch counts and could potentially lead to underdosing of reversal agents. Despite this, this reflected real-world practice variation, particularly during a period when the American Society of Anesthesiologists Task Force on Neuromuscular Blockade’s practice guidelines regarding the use of sugammadex were established and progressively adopted. Prior works have shown that clinical evaluation of paralysis assessment is overall poor with sensitivities ranging from 11 to 14% [21]. Despite this, the trial design accommodated anesthesia discretion for paralysis assessment irrespective of a patient’s allocation group and therefore applied to both randomized groups. Despite this, the consequences of misclassification may not be symmetric. Given neostigmine reversal is more dependent on adequate spontaneous recovery at the time of administration, with sugammadex being more effective across moderate and deeper rocuronium-induced blockade, if differential, this limitation would most likely bias recovery-related outcomes in favor of sugammadex. However, despite this possibility of non-differential impact, there were no difference between our groups. Third, as a single-center study, our findings may not be fully generalizable to other institutions, and the characteristics of our AWR cohort may differ from those of broader abdominal surgery populations. Lastly, our composite GI-2 endpoint, while clinically relevant, may not capture all aspects of gastrointestinal recovery.

## Conclusion

In this randomized controlled trial, sugammadex did not reduce time to gastrointestinal recovery, postoperative ileus incidence, or hospital length of stay compared to neostigmine/glycopyrrolate in patients undergoing complex abdominal wall reconstruction. Given the substantial cost differential between agents and the absence of demonstrated clinical benefit in this population, routine sugammadex use is not supported for complex abdominal wall reconstruction in relationship to post-operative gastrointestinal motility.

## Abbreviations

ACHQC: Abdominal Core Health Quality Collaborative
AWR: Abdominal wall reconstruction
EMR: Electronic Medical Record
ERAS: Enhanced Recovery After Surgery
GI-2: Gastrointestinal Recovery of Function
MME: Morphine Milligram Equivalents
REDCap: Research Electronic Data Capture
POI: Postoperative Ileus

## Declarations

### Clinical trial number

Clinicaltrials.gov; NCT05985343.
